# Methicillin-susceptible *Staphylococcus aureus* skin infections among military conscripts undergoing basic training in Bangkok, Thailand, in 2014

**DOI:** 10.1186/s13104-016-1989-3

**Published:** 2016-03-19

**Authors:** Thirapa Nivesvivat, Dusit Janthayanont, Mathirut Mungthin, Julphat Intarasuphit, Siriwan Paojinda, Kanya Phanitorn, Paijit Permpool, Saowapap Kasinant, Onuma Pengpinij, Parichart Yingprasert, Wanida Thaochelee, Ram Rangsin

**Affiliations:** Department of Military and Community Medicine, Phramongkutklao College of Medicine, 315, Ratchawithi Road, Ratchathewi, Bangkok, 10400 Thailand; Department of Outpatients, Phramongkutklao Hospital, Bangkok, Thailand; Department of Parasitology, Phramongkutklao College of Medicine, 315, Ratchawithi Road, Ratchathewi, Bangkok, 10400 Thailand; Division of Dermatology, Department of Internal Medicine, Phramongkutklao Hospital, Bangkok, Thailand; Infection Control Unit of Phramongkutklao Hospital, Bangkok, Thailand; Clinical Epidemiology Unit of Phramongkutklao Hospital, Bangkok, Thailand

**Keywords:** Methicillin-susceptible *Staphylococcus aureus*, Skin infection, Military, Outbreak

## Abstract

**Background:**

Skin and soft tissue infections are common among military conscripts undergoing close-contact training activities. On June 4, 2014, an outbreak of *Staphylococcus aureus* skin infection was reported among military conscripts undergoing basic training in Bangkok, Thailand. An investigation was performed to verify the outbreak and recommend future prevention and control strategies.

**Case presentation:**

The outbreak resulted in a rate of infection of 19.2 % and a fatality rate of 2.5 % (one death). All were Thai men aged 21.2 ± 1.0 years. Risk factors associated with infection were multiple erythematous papules and training in certain subunits. Randomly selected isolates were evaluated by pulsed field gel electrophoresis to confirm the clonal identity.

**Conclusions:**

This report confirms that *S. aureus* skin infection can be fatal. Our study highlights the role of military personnel in the early detection, prompt treatment, and containment of outbreaks of skin infection, as well as other health issues among conscripts.

## Background

On June 4, 2014, 25 conscripts were found to possess abscesses on their arms and elbows. This outbreak of infection resulted in one death. The Clinical Epidemiology Unit investigated other soldiers at the basic military camp on that day to determine the cause(s) of the infection and recommend appropriate prevention and control strategies. In April each year, approximately 9–10 % of men aged 21 years with a good health profile from all provinces are randomly selected to serve in the army, beginning with 10 weeks of basic military training. The reported outbreak occurred 2–6 weeks after training started, a time when training becomes more intensive.

### Epidemiological investigation

The descriptive data were collected on June 5, 2014. Of 213 recruits, 212 completed the self-reported questionnaire. The information in the questionnaire included demographic data, clinical manifestation, date of onset, number of lesions, and location(s) of lesions on their body. Demographic questions included age, sex, education, underlying diseases, and unit types. Clinical manifestations comprised (1) abscesses (2) multiple erythematous papules and (3) fever. Those reporting abscesses were considered as probable cases. The height and weight of the recruits were obtained from heat stroke screening data to calculate the body mass index (BMI) [1] and identify overweight or obese cases.

A case was defined as a person who was clinically diagnosed with an infectious skin lesion, including abscesses and furuncles, from mid-May to the first 2 weeks of June. The infecting bacteria were confirmed to be methicillin-susceptible *Staphylococcus aureus* (MSSA) using standard culturing techniques. Healthcare providers and recruits were interviewed about their daily activities, such as hand-to-hand combat training, the characteristics of physical training, and hygiene.

In the case of the deceased patient, his medical records, signs and symptoms, laboratory information, treatment, and cause of death were reconsidered.

### Laboratory investigation

Pus were collected from the sites of infection. The clinical isolates were sent for bacterial culture at the Division of Microbiology, Department of Clinical Pathology, Phramongkutklao Hospital. Standard bacteriological culturing by inoculation of blood agar was performed, as well as antibiotic susceptibility testing using the disk diffusion method. During our investigation, antibiotic susceptibilities to oxacillin, gentamicin, ciprofloxacin, levofloxacin, trimethoprim-sulfamethoxazole, chloramphenicol, tetracycline, clindamycin, fusidic acid, fosfomycin, linezolid, vancomycin, and erythromycin were determined. Isolates that were positive for *S. aureus* were forwarded to the Reference Laboratory of the National Institute of Health, Department of Medical Sciences, Ministry of Public Health, Thailand, where they were confirmed to be *S. aureus* by standard culture techniques. The PCR technique adopted by the National Institute of Health accompanied sensitivity analysis by means of disk diffusion (Kirby Bauer) and E-test (vancomycin) assays to differentiate MSSA and methicillin-resistant *S. aureus* (MRSA) [2]. We randomly selected isolates to be further evaluated by pulsed field gel electrophoresis (PFGE) [3] to confirm the clonal identity.

### Environmental investigation

Environmental samples, comprising combat uniforms, bathing water, and bath reservoirs shared among recruits were collected for bacterial culture using blood agar. The morning after the investigation was completed, the investigation team prescribed antibiotics for all cases.

### Statistical analysis

The case rates were calculated both within each unit and for the whole battalion. Descriptive data were gathered by means of a questionnaire. These data were used to find an association between cases and risk factors. Case fatality was calculated.

The methodologies employed were univariate and multivariate analyses. Using STATA software, the methodologies resulted in crude odds ratio and adjusted odds ratio, respectively, and 95 % confidence intervals. The variables with significant *p* values less than 0.05 were further considered.

Ethical approval was obtained from the Institutional Review Board of the Royal Thai Army Medical Department. Confidentiality was maintained throughout and after the study. Names were not included on the questionnaire for confidentiality reasons. The written informed consent for publication of the clinical details from the next of kin of the deceased patient was also obtained.

## Case presentation

A 21-year-old Thai male conscript was admitted to the hospital after fainting with urinary and stool incontinence during combat training on June 4, 2014. He was primarily assumed to be suffering from heat syncope. He was a previously healthy man from subunit number 2, who was 20 years old with a BMI of 21.4 kg/m^2^. Six days before being admitted, abscesses were detected on both arms and elbows and the military camp prescribed oral amoxicillin. Three days later, he developed a high fever, nausea, and vomiting. The Emergency Department examined his vital signs, revealing a body temperature at 38 °C, dyspnea and tachypnea (30/min), tachycardia (140/min), and a blood pressure of 130/80 mmHg. Later, his blood pressure dropped to 85/50 mmHg. The doctor administered normal saline infusion, accessed the central venous line for inotropic drugs to increase his blood pressure, and gave broad-spectrum antibiotics. Ceftriaxone (2 g per day) and clindamycin (600 mg every 8 h) were administered intravenously. The conscript was then sent to the intensive care unit, where he remained conscious. Physical examination revealed multiple ruptured abscesses on an erythematous edematous base of the left elbow. Laboratory tests revealed a raised level of creatinine phosphokinase (10 times higher than normal), as well as an electrolyte imbalance. Liver and renal panels were also found to be abnormal. Urinalysis showed hematuria and pyuria. The pus culture collected from his elbow and hemoculture indicated MSSA infection. Despite prompt treatment, the patient passed away.

**Fig. 1:**
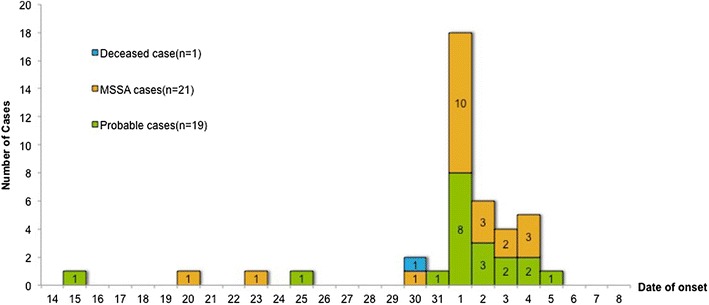
Epidemic curve of skin abscesses in conscripts by date of onset (n = 41)

A case was defined as a person who was clinically diagnosed with one or more abscesses from mid-May to the first 2 weeks of June. Of 213 recruits, 41 (19.2 %), including the deceased patient, were documented as contracting the infection, which was used as the case rate for the whole unit. The index case is thought to have occurred on May 15, 2014 (Fig. 1). Approximately 2 weeks later, four cases were reported from May 30 to 31. On June 1, the number of patients abruptly rose to 17 cases (41.5 %) in 1 day, reaching the highest peak for skin infections. The case fatality rate was 2.4 % (1/41).

All conscripts at this facility were male, with a mean age of 21.2 ± 1.0 years. They were divided into four subunits in the same facility. The clinical manifestations included abscesses (19.2 %), multiple erythematous papules (39.0 %), and fever (16.9 %). The lesions were located on elbows (46.3 %), shoulders (24.4 %), and legs (14.6 %).

The percentage of new conscripts who reported multiple erythematous papules were 51.9, 37.3, 32.7, and 39.6 % for subunits 1, 2, 3, and 4, respectively (*p* = 0.211). While the percentage of conscripts who displayed abscesses were 37.0, 31.4, 7.3, and 1.9 % for subunits 1, 2, 3, and 4, respectively (*p* < 0.01) (Table 1). Adjusted odds ratios for belonging to subunits 1 and 2 were 32.6 (4.1–260.6, 95 % CI) and 26.9 (3.3–218.2, 95 % CI) respectively. Multiple erythematous papules were significantly associated (*p* < 0.01) with abscesses. The adjusted odds ratio was 3.4 (1.5–7.5, 95 % CI) (Table 2).

**Table 1:** Characteristics of all conscripts during basic military training in Bangkok, Thailand (n = 212)

Demographic data	Total (%)
Age (mean ± SD)	21.2 (±1.0)
20	19 (8.9)
21	156 (73.2)
22	21 (9.9)
23	10 (4.7)
≥24	7 (3.3)
Education	
Bachelor’s	8 (4)
High vocational certificate	14 (7)
Vocational certificate	22 (10.9)
High school	106 (52.7)
Secondary school	51 (25.4)
Subunit	
1	54 (25.4)
2	51 (23.9)
3	55 (25.8)
4	53 (24.9)
Weight (kg) (mean ± SD)	64.3 ± 11.4
Height (cm) (mean ± SD)	170.2 ± 6.0
BMI (kg/m^​2^) (mean ± SD)	22.2 ± 3.6
Underweight <18.5	27 (12.7)
Normal range (18.5–22.9)	119 (55.9)
At risk of obesity (23–24.9)	29 (13.6)
Obese I (25–29.9)	29 (13.6)
Obese II (≥30)	9 (4.2)
Symptoms	
Abscess	41 (19.2)
Number of abscess lesions (mean ± SD)	2.2 ± 2
Multiple erythematous papules	86 (39.0)

**Table 2:** Univariate and multivariate analysis for risk factors of acquiring skin abscesses

Risk factors	Total (%)	Case (%)	*P* value	Crude OR (95 % CI)	*P* value	Adjusted OR (95 % CI)
Age (mean ± SD)	21.2 (±1.0)	21.0 (±1.0)	0.033	0.5 (0.3–0.9)	0.036	0.5 (0.3–1.0)
Education						
Bachelor	8 (4)	3 (37.5)		1		
High vocational certificate	14 (7)	4 (28.6)	0.666	0.7 (0.1–4.2)		
Vocational certificate	22 (10.9)	5 (22.7)	0.423	0.5 (0.1–2.8)		
High school	106 (52.7)	19 (17.9)	0.191	0.4 (0.1–1.7)		
Secondary school	51 (25.4)	7 (13.7)	0.112	0.3 (0.1–1.4)		
Subunit						
1	54 (25.4)	20 (37)	0.001	30.6 (3.9–238.6)	0.001	32.6 (4.1–260.6)
2	51 (23.9)	16 (31.4)	0.003	23.8 (3.0–187.5)	0.002	26.9 (3.3–218.2)
3	55 (25.8)	4 (7.3)	0.216	4.1 (0.4–37.7)	0.178	4.6 (0.5–44.1)
4	53 (24.9)	1 (1.9)		1		
BMI (kg/m^​2^)						
Underweight <18.5	27 (12.7)	7 (25.9)	0.275	1.7 (0.6–4.6)		
Normal range (18.5–22.9)	119 (55.9)	20 (16.8)		1		
At risk of obesity (23–24.9)	29 (13.6)	5 (17.2)	0.955	1.0 (0.4–3.0)		
Obese I (25–29.9)	29 (13.6)	7 (24.1)	0.362	1.6 (0.6–4.2)		
Obese II (≥30)	9 (4.2)	2 (22.2)	0.679	1.4 (0.3–7.3)		
Multiple erythematous papules						
Yes	86 (39.0)	27 (31.4)	<0.01	3.7 (1.8–7.6)	<0.01	3.4 (1.5–7.5)
No	127 (59.6)	14 (11)		1		

The data gathered from interviews with healthcare providers and conscripts revealed that 1–2 weeks before the outbreak, part of the training of subunit 1 and 2 conscripts involved low crawling on a concrete floor.

A dermatologist from the investigation team was able to identify various kinds of skin lesions including multiple erythematous papules, multiple discrete small dermal pustules with surrounding erythema, and skin abscesses. Pus from those suffering from abscesses was collected on June 5, 2014. From 29 clinical cultures, 22 (75.9 %), including one blood culture of the deceased patient, grew MSSA isolates. Among all 22 subjects who were positive for MSSA culture (Table 3): one person died; 50.0 % reported fever; 86 % were from subunits 1 or 2, and all had a history of skin abscess (es). In addition, three cultures (10.3 %) were positive for *Bacillus* spp. and one culture (3.4 %) was positive for the following organisms: *Corynebacterium* spp*., Streptococcus pyogenes, Klebsiella pneumoniae* (*ESBL*-*producing strain*)*, Enterococcus faecalis, Citrobacter* spp*., Acinetobacter baumannii,* and *Pseudomonas stutzeri.*

**Table 3:** *Staphylococcus aureus* susceptibility test results for all available isolates

No. of isolates	Antimicrobial, no. of isolates tested (%)								
	Oxa	Gen	Cip	Co-tri	Chlo	Tet	Cli	Van	Ery
22	22 (100.0 %)	22 (100.0 %)	22 (100.0 %)	22 (100 %)	14 (63.6 %)	4 (18.1 %)	19 (86.6 %)	22 (100.0 %)	22 (100.0 %)

*oxa* oxacillin, *gen* gentamicin, *cip* ciprofloxacin, *co*-*tri* trimethoprim sulfamethoxazole, *chlo* chloramphenicol, *tet* tetracycline, *cli* clindamycin, *van* vancomycin, *ery* erythromycin

**Fig. 2:**
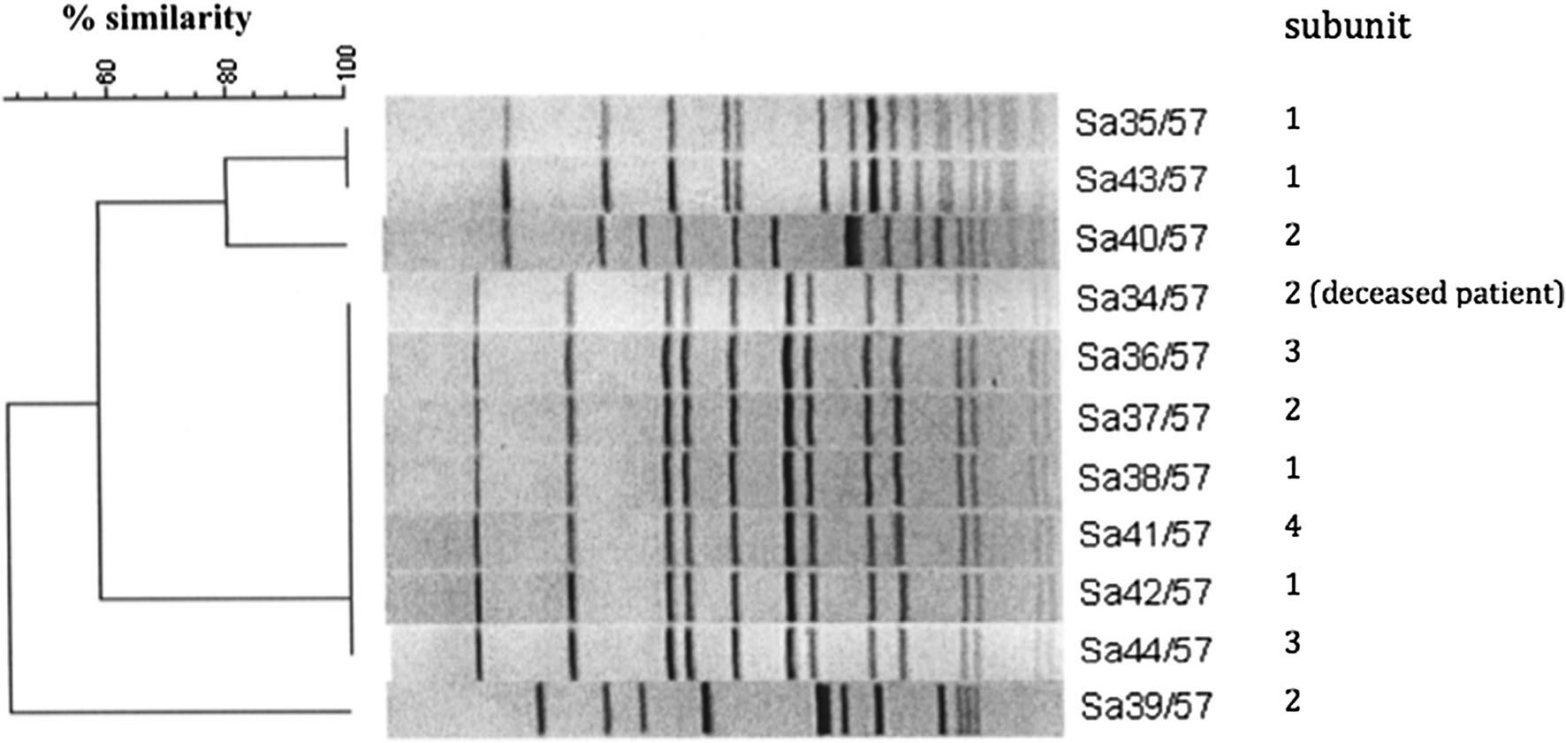
Dendrogram of methicillin-susceptible *Staphylococcus aureus*, as analyzed by the BioNumerics software

A total of 11 randomly selected isolates, including one isolate collected from blood culture of the deceased patient, were evaluated by PFGE to confirm clonal identity revealing four genetic patterns (Fig. 2). Sa 35/37 and Sa 43/57 had 100 % genetic similarity. Sa 34/57 (from the deceased patient), Sa 36/57, Sa 37/57, Sa 38/57, Sa 41/57, Sa 42/57 and Sa 44/57 also showed 100 % genetic similarity. Sa 39/57 and Sa 40/57 had their own genetic patterns. All environmental swabs were negative for bacterial cultures. The morning after the investigation was completed, dicloxacillin (250 mg per oral qid) was prescribed for all cases.

## Conclusions

Prevalence of MSSA nasal colonization among healthy young adults in Thailand has been reported to be 15 %, while prevalence of MRSA nasal carriage was 1 % [4]. Patients infected with MSSA were also reportedly more prevalent than patients infected with MRSA [5]. Among soldiers in the US army, a study reported that 38 % were colonized with MSSA and 3.9 % were colonized with MRSA [6].

Investigations revealed that the pathogen responsible for abscesses in military populations in this present study was MSSA. This organism has generally been reported to be less harmful than MRSA, and more susceptible to antimicrobial therapy [7]. However, here we report a conscript who displayed abscesses and consequently died of MSSA septic shock. The cultures from skin tissue and hemoculture were positive for MSSA. Considering the laboratory results and the fact that prompt treatment was initiated but was ineffective, the patient most likely suffered septic shock and multiorgan failure.

Previous studies have identified the prevalence and risk factors of community-associated methicillin-resistant *S. aureus* (CA-MRSA) [8–10]. Although the risk factors associated with acquiring CA-MRSA and MSSA infection are similar, the information regarding MSSA infection is limited [8, 11–13]. Inadequate hygiene and physical trauma are likely to lead to the emergence of MRSA infection among military trainees [12, 13]. The higher case rates observed for subunits 1 and 2 compared with subunits 3 and 4 in our study might be explained by the training activity involving low crawling on a concrete floor prior to the onset of abscess skin lesions among conscripts in subunits 1 and 2, while conscripts in subunits 3 and 4 did not undergo any such activity during the same period. Low crawl training often causes skin abrasions on elbows and 43 % of cases had abscesses on their elbows.

Furthermore, multiple erythematous papules are another important risk factor associated with abscess development. A recent study revealed that control of eczema can reduce the chance of *S. aureus* infection [14] and compromised skin integrity has been found to be associated with CA-MSSA infection [15].

A report of skin infection caused by MSSA or MRSA revealed the Panton-Valentine leukocidin (PVL) gene as a virulence factor [6]. It is possible that this outbreak of MSSA skin infection was associated with the PVL gene. In one study, some MSSA isolates from blood culture or skin lesions were found to be positive for the PVL gene [5].

*Staphylococcus aureus* usually colonizes the anterior nares [16, 17] and may be transmitted to clothes and other parts of the skin by touching. PFGE revealed that MSSA isolated from the conscripts exhibited four DNA patterns, one of which was shared by 7 of 11 clinical isolates. Among the other four isolates, two shared an identical DNA pattern. However, different DNA patterns may be observed for the same strain, and similar DNA patterns may not necessarily indicate the same family of isolates [18]. The varying health status and behavioral activities of individuals in a community mean that some pathogens may be transmitted into the environment and passed on to others. Researchers have advocated that clones identified by PFGE are indistinguishable from outbreak isolates and suggest person-to-person transmission [13].

In summary, we identified an outbreak of MSSA infection among new military conscripts in a crowded environment during basic training. The risk factors associated with infection were identified as training exercises that involved crawling on a hard floor often resulting in skin abrasions, and the sharing of towels. Delayed diagnosis and ineffective antibiotic treatment led to severe infection and fatality, for this normally treatable condition. We recommend that a survey of *Staphylococcus* carriage among military camps be undertaken by the military medical agency to prevent similar outbreaks in the future.

### Consent

Patient consent forms were waived because this was an outbreak investigation and the study was approved by the royal Thai army medical department institutional review board.
